# Disease Activity, Occupational Participation, and Quality of Life for Individuals with and without Severe Fatigue in Ankylosing Spondylitis

**DOI:** 10.1155/2019/3027280

**Published:** 2019-07-01

**Authors:** Deirdre Connolly, Clodagh Fitzpatrick, Finbar O'Shea

**Affiliations:** ^1^Discipline of Occupational Therapy, School of Medicine, Trinity College Dublin, Dublin 2, Ireland; ^2^Rheumatology Department, St. James Hospital, James' Street, Dublin 8, Ireland

## Abstract

**Background:**

Fatigue is one of the most frequently reported symptoms by individuals with ankylosing spondylitis. However, it is often overlooked clinically and in research. Literature researching the impact of severe fatigue on occupational participation in ankylosing spondylitis is limited. Therefore, the aim of this research was to explore the impact of severe fatigue on occupational participation, disease activity, and quality of life in people with AS.

**Methods:**

A sequential exploratory mixed method study design was used in this study. Self-reported questionnaires gathered quantitative data which were analysed with descriptive and inferential statistics. Qualitative data were generated through semistructured interviews and analysed using a content analysis approach.

**Results:**

Fifty individuals with AS completed all study questionnaires. Participants had a mean age of 46.5 years; 72% were men with a mean disease duration of 14.5 years. High fatigue was reported by 38% of participants using the Multidimensional Assessment of Fatigue (MAF). Fatigue was significantly associated with lower occupational participation (*p* = 0.018), higher disease activity (*p* < 0.001), higher pain (*p* < 0.001), reduced physical capacity (*p* = 0.018), lower quality of life (*p* < 0.001), and lower global well-being (*p* < 0.001). There were significant differences between those with low and high fatigue levels for occupational participation (*p* = 0.007), disease activity (*p* < 0.001), physical capacity (*p* = 0.015), pain (*p* < 0.001), and quality of life (*p* < 0.001). Participants discussed the impact of fatigue on productivity and leisure. They also discussed a range of strategies for managing their fatigue but reported a lack of education from health professionals on managing this symptom.

**Conclusion:**

Severe fatigue is a prevalent symptom for individuals with ankylosing spondylitis and results in reduced occupational participation in productivity and leisure. Early fatigue management interventions may reduce the occupational participation impact of this symptom for individuals with ankylosing spondylitis.

## 1. Introduction

Ankylosing spondylitis (AS) is a chronic inflammatory rheumatic condition of the spine with disease onset usually occurring between late adolescence and early adulthood [1, 2]. Previously, it was thought that AS occurred mostly in young men but recent research has found a 2-3 : 1 ratio of men to women [3]. The main symptoms of AS are pain, stiffness (especially in the morning), reduced mobility, decreased function, and fatigue [4]. The pain and mobility challenges due to AS impact on daily functioning and result in reduced physical activity and quality of life [2]. However, exactly how AS impacts on engagement in daily activities requires further exploration [5].

Medical management of AS focuses on reducing and controlling pain and inflammation and preserving physical functioning and quality of life while also trying to minimise damage to joints caused by the disease process [2]. Biologic medications are the most common medical treatment approach used in AS to reduce pain and stiffness and improve daily functioning [6, 7]. However, the European League Against Rheumatism states that AS requires a multidisciplinary approach to intervention with optimal interventions combining medical and nonpharmacological management [8]. This combined approach optimizes participation in daily activities and health-related quality of life.

Fatigue has been identified as a prominent symptom of AS and is now the third most commonly reported symptom after stiffness and pain [9, 10, 11, 12]. Schneeberger et al. [13] reported that fatigue is present in up to 73.4% of individuals with AS compared to 30.5% in a non-AS controlled comparison group. For individuals with AS, fatigue impacts on emotions, functional ability, quality of life, and ability to maintain employment [10, 13, 14].

Although no definite causes have yet been identified for fatigue in AS, a number of factors are believed to be associated with increased fatigue. These include disease activity, pain, stiffness, low mood, and lifestyle factors such as reduced physical activity, poor diet, and disrupted sleep [13, 15]. Pharmacological interventions for fatigue include low doses of amitriptyline [16]. Nonpharmacological interventions for fatigue for individuals with rheumatic diseases include energy management education, cognitive behavioural therapy, and exercise [14].

Occupational therapists are often the profession to provide fatigue management interventions and education to individuals with chronic diseases who experience fatigue [17]. However, previous research in fatigue in rheumatic diseases has identified variations in patterns of fatigue and factors that increase fatigue. Therefore, in order to tailor fatigue management interventions for individuals with AS, a greater understanding is needed on the specific impact of severe fatigue on occupational participation and factors associated with fatigue [18]. The aim of this study was to (i) identify prevalence of severe fatigue in a cohort of people attending an AS clinic; (ii) examine differences in those with severe fatigue for disease variables, occupational participation, and quality of life; and (iii) explore experiences of fatigue and management strategies.

## 2. Methods

### 2.1. Participants

Participants were recruited from a dedicated weekly AS clinic in an urban hospital using convenience sampling [19]. Clinic attendees were invited to participate if they met the inclusion criteria of over 18 years of age with a definite diagnosis of AS. Individuals who had a diagnosis of any other type of rheumatic disease or other chronic diseases associated with increased fatigue were excluded. During the eight-month data collection period, 58 people met the inclusion criteria of which 50 people completed study questionnaires. Those who completed the questionnaires were invited to indicate at the end of the questionnaire if they would participate in an interview to discuss their fatigue in more detail. Thirty-two individuals agreed to participate in an interview. Those who did not participate stated they had no fatigue or were not interested in participating in the study. Ethical approval was received from the recruiting hospital's research ethics committee.

### 2.2. Data Collection

Three fatigue measures were used in this study. Two of the scales, the Fatigue Severity Scale (FSS) [20] and the Bath Ankylosing Spondylitis Disease Activity Index (BASDAI) [21] fatigue single item, are unidimensional measures which assess the presence and severity of fatigue. The third scale, Multidimensional Assessment of Fatigue (MAF) [22], measures the impact of fatigue on a range of activities of daily living.

#### 2.2.1. Fatigue Severity Scale (FSS) [20]

The FSS is a 9-item self-report fatigue scale that measures the impact of fatigue on everyday activities. It generates a global score from 0-7, with a score of four and over indicating significant fatigue [20]. The FSS has been found to demonstrate good internal consistency, validity, and reliability and is sensitive to change [23]. It has been used in previous research with people with AS and other rheumatic conditions [24].

#### 2.2.2. Bath Ankylosing Spondylitis Disease Activity Index (BASDAI)

The BASDAI is an AS-specific measure designed to assess an individual's experience of disease activity over the past week [21]. It consists of six 10-point scales, assessing different aspects of disease activity in the past week ranging from 0 (none) to 10 (very severe). The total score is the mean of the six items with low scores indicating less disease activity [25]. A score of 4 or more indicates high disease activity [21]. The BASDAI has proven to show good construct and content validity, along with having good test-retest reliability [26].

The single-item fatigue visual analogue scale (VAS) on the Bath Ankylosing Spondylitis Disease Activity Index (BASDAI) is measured on a scale of 0-10 (none to very severe) [21]. Individuals are asked to rate their fatigue over the past week. A score of five or above indicates significant fatigue. Fatigue VAS are valid measures of fatigue and are recommended for a global measure of fatigue [27].

#### 2.2.3. Multidimensional Assessment of Fatigue (MAF) [22]

The MAF is a 16-item self-report measure used to assess the multiple aspects of fatigue. The MAF generates a global score the Global Fatigue Index (GFI) from the first 15 items of severity, distress, impact on daily activities, and timing of fatigue. The scores range from 1 (no fatigue) to 50 (severe fatigue) [27]. A cut-off score of 21 and over in the MAF-GFI indicates high fatigue levels [22]. The MAF has high internal consistency, criterion and construct validity, and reliability and is sensitive to change [27].

#### 2.2.4. Frenchay Activities Index (FAI) [28]

The FAI is a 15-item self-report questionnaire that consists of three subscales of domestic activities, leisure/work, and outdoor activities [28]. The questions are scored on a four-point scale from 0 (never participate) to 3 (daily/weekly/greater than 30 hours per week participation). The subcategories are scored on a scale of 0-15 and the total score ranges from 0-45. A low score indicates low levels of activity participation and a high score indicates greater activity participation frequency. The FAI has strong criterion and construct validity and excellent test-retest reliability in community-dwelling populations [29].

#### 2.2.5. Patient Global Assessment of Disease Activity (PtGA)

The PtGA is a single-item 0-10 scale designed to measure the overall impact of AS on the individual at a point in time. Higher scores indicate higher disease activity [30]. The PtGA has strong test-retest reliability, validity, and response to changes [26].

#### 2.2.6. Bath Ankylosing Spondylitis Functional Index (BASFI) [31]

The BASFI is a 10-item self-report questionnaire that measures an individual's ability in physical movements such as bending, reaching, turning, and climbing steps [31]. These movements are core components of daily activities that are often affected by AS [32]. Each item is scored from 0 (easy) to 10 (impossible) with a total score generated by calculating the mean of the 10 items [31]. The BASFI has acceptable test-retest reliability and internal validity and is sensitive to change [26].

#### 2.2.7. Pain Numeric Rating Scale (NRS) [33]

Pain (total and nocturnal) was assessed using a numerical rating scale ranging from 0 (no pain) to 10 (unbearable pain) with higher scores indicating more severe levels of pain [33]. This test has high test-retest reliability in individuals with rheumatic diseases and strong construct validity amongst rheumatic diseases and other chronic pain conditions [34].

#### 2.2.8. Ankylosing Spondylitis Quality of Life Questionnaire (ASQoL) [35]

The ASQoL measures the impact of AS on health-related quality of life. It is an 18-item binary response scale. Items include impact of AS on sleep, mood, motivation, coping, activities of daily living, independence, relationships, and social life. Scores range from 0-18 with higher scores indicating poorer quality of life [35]. The ASQoL has good responsiveness, internal and test-retest reliability, and construct validity [26].

#### 2.2.9. Bath Ankylosing Spondylitis Patient Global Score (BAS-G) [36]

The BAS-G assesses a person's overall sense of well-being in relation to disease activity in AS. The BAS-G consists of two 10-point scale questions (0 no effect to 10 very severe effect) related to well-being over the past week and the past six months. The mean of the two scores is the final score with the scores ranging from 0 (no effect of disease on well-being) to 10 (worst effect disease on well-being) [36]. The BAS-G has high test-retest reliability; it is sensitive to change and sustains face, predictive, and construct validity [26].

Qualitative data were collected through semistructured interviews. Individual interviews are the most frequently used data collection method in qualitative studies [37]. An interview guide asked participants to describe their fatigue and to identify if and how it impacted on occupational participation. The interview guide was forwarded to participants prior to their interview to enable them to review the interview questions prior to their interview [38]. All interviews were completed by one of the researchers (CF) and were carried out following completion of the questionnaires in a separate meeting.

## 3. Data Analysis

Data were analysed using Statistical Package for the Social Science (SPSS) version 20. Descriptive statistical analysis was carried out to test for trends and frequencies [39]. Nonparametric one-tailed correlational statistical analysis was used to test relationships between variables, and the Mann-Whitney *U* test examined differences between variables [40].

All interviews were transcribed verbatim and transferred to NVivo computer software for data analysis. Content analysis was used to guide analysis of the qualitative data. The aim of content analysis is to provide a condensed and broad description of the study phenomenon [41]. Two of the authors coded the transcripts separately and then came together to compare codes across all interviews. Differences in codes were discussed, and where there was disagreement, codes were agreed on and renamed and/or new codes developed and applied across all transcripts. On completion of the coding process, codes were grouped into categories of themes based on the study aims [42]. Copies of transcripts and a summary of the analysis were made available to participants for review to ensure that interpretation of the data was consistent with experiences of the participants [43]. No changes were made by participants to their transcripts or the analysis summaries.

## 4. Results

### 4.1. Quantitative

Fifty individuals with a confirmed diagnosis of AS participated in this study with 36 men and 14 women. The mean age of participants was 46.5 years, ranging from 26 to 76 years.  Table 1 gives the demographic and disease-related information of the study cohort. Participants had their disease for a mean number of 14.5 years (SD ± 12.1).

The mean FSS and BASDAI fatigue item scores were 4.2 (SD ± 1.6) and 5.6 (SD ± 2.4), respectively, with 64% of individuals with significant fatigue on the FSS and 66% on the BASDAI (Table 2). The mean score for the MAF-GFI was 18.2 (SD ± 7.7) with 38% above the significant fatigue cut-off score of 21. Overall, study participants engaged frequently in activities with a mean score of 31.9 out of 45 in the FAI total score (Table 2). The lowest scoring category was in leisure/work with a mean score of 9.7 out of 15. For self-reported disease activity, the mean scores on the BASDAI and PtGA were just above the high disease activity cut-off scores of four with a medium to high impact of AS on physical ability (BASFI), pain (total and nocturnal), quality of life, and global well-being (ASQoL and BAS-G).

Spearman rank correlational analyses tested for relationships between fatigue, activity levels, and disease-specific variables (disease activity, physical ability, pain, and quality of life) (Table 3). The MAF-GI and BASDAI fatigue items were significantly associated with all study measures.

As the MAF-GFI was significantly associated with all variables and it measures the impact of fatigue on daily activities, this measure was used to examine differences between individuals with high and low fatigue for activity participation, disease activity, physical abilities, pain, quality of life, and global well-being. There were significant differences in all study measures between those with high and low fatigue levels (Table 4). There were no significant differences between high and low MAF-GFI scores for age (*p* = 0.746), disease duration (*p* = 0.702), education levels (*p* = 0.946), and employment status (*p* = 0.814).

### 4.2. Qualitative

Thirty-two people agreed to participate in an interview exploring the impact of fatigue on occupational participation. Of these, 19 people participated in the interviews. The remaining 13 were either unavailable for interview or not responding to the research team when contacted to arrange an interview despite repeated efforts.  Table 5 provides the demographic characteristics and employment status of interview participants.

Four themes were identified from analysis of the qualitative data: (i) impact of fatigue on productivity, (ii) impact of fatigue on social participation, (iii) fatigue management strategies, and (iv) fatigue management education.

#### 4.2.1. Impact on Productivity

Eleven of those interviewed were in employment, and they discussed the impact of fatigue on their work performance particularly in relation to cognitive skills such as concentration:

“A lot of the work I do involves reading documents and synthsising and summarising them and you need concentration to do that. And sometimes I find that where ordinarily if you're focused you'd only have to read over a document once whereas I'd have to maybe read it two or three times for it to sink in.” (P52)

Four participants discussed having to work longer hours during the week and at weekends in order to get all their works completed:

“I find that my working hours are extending into the evenings and sometimes into the weekend in order for me to meet the different requirements of the work that I'm doing at any particular time. So when that happens it impacts on other aspects of my life in terms of my fitness and in terms of getting away from work. And so it's kind of a catch twenty-two.” (P52)

One participant whose work involved a considerable amount of time driving discussed the impact of this on stiffness and fatigue:

“I find long periods in the car very hard physically and you're fatigued. You get so tired. The longest I could travel in a car now would be 45 minutes to an hour, and I'd have to get out - without a doubt I'd have to get out and walk around.” (P15)

Over half of the participants discussed modifying their work hours because of fatigue. For example:

“I'm just finding it too tiring now. So I want to go back to doing part-time because full-time is too much for me.” (P13)

The impact of fatigue on housework was also discussed:

“I could be doing just a few things around the house and I'd feel tired. It wouldn't last very long, it works its way in and then I have to just stop and relax. Then the pain will ease off a little and I don't feel as tired.” (P25).

A female participant discussed the impact of grocery shopping on her fatigue and the difficulty she experienced when shopping without her husband:

“If he (participant's husband) has to work, and I have to go and do it on my own, it's twice as hard because then of course I have to do the loading into the trolley and unloading it onto the belt and then loading it back off. Whereas, when we do it together it's shared. It takes a lot out of you!” (P3)

#### 4.2.2. Impact of Fatigue on Social Participation

The majority of participants discussed opting out of social activities because of their fatigue and needing to conserve their energy for work. One participant described how this impacted on his weekend social activities:

“Even at the weekend my girlfriend will say to me “Will we go out?”…and I'm just “Oh no - let's just sit in and get a Chinese or something.” I just couldn't be bothered. It does have a huge impact on your social life, without a doubt, because you'd just prefer to be at home because you're so tired.” P15

One participant described feeling relieved when a social event was cancelled: “When it comes to going out, and somebody cancels, the relief you have because you're tired - you're thinking ‘great I can have an early night'.” (P28)

The majority of participants identified how being fatigued after work results in reduced energy for social activities:

“I'm tired when I get home from work and if I'm asked to go out somewhere I dread it! I think “Oh God” and it's a late night. When I'm in a routine my body needs the rest after a day's work. If it's an evening out I can't go past 12 o' clock! I know that sounds terrible, but I'm just tired at that stage, I'm aching.” (P13)

#### 4.2.3. Management Strategies

Study participants identified various strategies they used to manage their fatigue such as pacing daily activities, planning their routines according to their energy levels, and making certain lifestyle choices.

Fourteen participants discussed how they pace their daily activities in order to reduce the impact of fatigue. For example:

“I try to take it easy… not to do so much during the day or to pack too much into the week and I'm still learning how to do that.” (P18)

“And you learn how to deal with pain and the same thing with fatigue because I love doing the gardening but I know when to stop have a bit of a break and then go back out and do it again. So, it's more about how you deal with these issues as opposed to letting them get on top of you.” (P34)

One participant uses a diary to plan his weekly schedule:

“I use my diary to set things out but I still have a tendency to do too many things and then you're really shattered.” P18

One of the participants who worked in construction discussed how working outside in extreme weather conditions affected his back pain and subsequently increased his fatigue. He managed this by negotiating a change in his work duties with his colleagues:

“I had to ask, because if not I would have lost my job. So I said to my colleagues “we're going to have to do something about this!” So I set out an agreement with the lads that I do all the work inside and they share the work outside and they're pretty happy with that.” (P12)

Many participants discussed how early in their disease trajectory they tended to ignore their fatigue and to carry on with their activities regardless of how much fatigue they experienced. For example:

“I thought that the best way for me to manage my fatigue was to ignore it and that eventually it would go away and that it just wouldn't be a problem for me.” (P18)

However, this participant described how when we decided to communicate with family members and work colleagues about his fatigue that this helped him to accept the limitations caused by fatigue:

“It's been helpful to speak out about it and to say “I do have some limitations” and it's okay to say that about myself because I used to get very annoyed when I couldn't do things, but not anymore.” (P18)

Changing attitudes towards fatigue was also identified as important:

“It's just one of these things that you have to learn to live with or else let it get to you.” (P34)

Six participants identified that taking regular exercise was an effective method for managing fatigue. For example:

“Get some sort of exercise, because I know I have to keep moving. You just can't be a lay-about when you have this …. you just have to keep on the move.” (P36)

“Your fatigued, you're feeling tired, you have your dinner in the evening and there's a lack of enthusiasm and motivation about putting on a pair of trainers and walking out the door and walking a few blocks or whatever it might be. But you need to discipline that and to watch that.” (P52)

The majority of participants discussed how taking rests during the day was helpful in managing fatigue. One participant described how taking a short rest can help her to complete an activity if having difficulty:

“I have to close my eyes. I suppose it's a case of zoning out. I don't think I'm officially asleep. I'm quite conscious of not going into sleep mode. But you need that rest, that down time.” (P35)

“When I'm tired, and my wife wants me to do something, I just say to her ‘I'm wrecked just let me have a rest for a while'. Then once I have that rest I'm grand again!” (P34)

#### 4.2.4. Fatigue Management Education

Almost all participants identified a lack of education from health professionals regarding fatigue. They reported mixed responses to fatigue when they tried to bring it up during hospital appointments.

“I don't think it gets enough attention.” (P51)

One participant reported that fatigue was often a minor aspect in educational material on how to manage symptoms of AS. “Yeah but maybe there's just a lack of knowledge about it around. I've been given pamphlets about arthritis and the fatigue element is either very minor or never discussed on it.” (P15)

One of the interview participants believed that this was due to people with AS not discussing their fatigue with their health professionals:

“It's a huge aspect of the disease and I wouldn't blame medical people that it's under rated. I think the patients themselves probably don't articulate it enough. No one in the medical profession has asked me about my fatigue, but by the same token, I haven't brought it up as an issue.” (P52)

As a result of not having received any formal education on fatigue, the majority of participants reported that most of their management strategies were self-developed:

“It's all just trial and error. You pick up on things as you go along, you just pick up on things day to day.” (P11)

## 5. Discussion

The majority of the participants in this study experienced significant fatigue as indicated by the FSS scores and the fatigue section of the BASDAI. This aligns with previous research which identified fatigue in up to 74% of individuals with AS [13]. Those with severe fatigue had significantly higher scores in all study measures including disease activity, pain, activity participation, and quality of life than those without sever fatigue. The qualitative findings in this study indicated that occupational participation levels were most frequently reduced in work and social activities. In work, fatigue impacted on physical and cognitive abilities which resulted in longer working hours to complete work duties or swapping work tasks with colleagues to those which are less physically demanding. There appeared to be a lack of education from health professionals on fatigue management.

There were significant relationships between fatigue (as measured by the BASDAI fatigue item and the MAF) and self-reported disease activity, physical capacity, pain, quality of life, and well-being. This demonstrates the relationship between these variables and how changes in clinical aspects of the disease and in fatigue can impact on each other. These significant relationships correspond with previous research examining relationships between fatigue, disease activity, and quality of life variables [4, 5, 13, 14]. On testing differences between those with and without severe fatigue, individuals with severe fatigue had statistically significantly higher disease activity, lower levels of occupational participation, and poorer quality of life. This corresponds with previous research [5, 14] and demonstrates the impact of severe fatigue across many domains for individuals with severe fatigue. These findings support the need to provide fatigue management interventions which focus both on managing symptoms of AS in addition to managing the impact of fatigue on productivity-related and leisure occupations.

On examining occupational participation, the FAI category of “leisure/work” had the lowest rates of participation with statistically significant differences in the frequency of participation in all categories of the FAI between those with and without severe fatigue. Leisure participation was discussed by interview participants who reported withdrawing from social activities because of fatigue. Low levels of satisfaction with participation in social roles were also identified by van Genderen et al. [44]. Participation in leisure activities is considered a significant predictor of quality of life [10, 11], and in this current study, there were significant differences in quality of life between those with high and low fatigue. This therefore supports the need for occupational therapists to provide strategies to individuals with high levels of fatigue to maintain engagement in valued leisure occupations.

The BASFI measures physical abilities such as bending, reaching, turning, and standing. In this study, physical ability was significantly related to fatigue and those with severe fatigue had significantly more difficulty with these movements. In their interviews, some participants identified housework and shopping as increasing their fatigue. These types of activities involve sustained reaching, bending, and standing which perhaps explains the reported difficulties with housework. This aligns with previous research which also found that AS limits individuals' ability to participate in certain home-based activities [11, 16, 44]. Education regarding joint protection and advice on rearranging home environments may assist with reducing the need for excessive bending and reaching to complete housework and shopping activities.

Sixty percent of study participants were working, and this was an area discussed by interview participants as resulting in considerable fatigue. Participants discussed different requirements of their work such as driving and cognitive skills of concentration and memory, being impacted by fatigue. Other studies have explored the impact of AS on work and have reported that individuals with AS are three times more likely to resign from work than the general population [45]. Work-focused self-management interventions result in significant improvements in work performance pain, fatigue, and mood [46]. This indicates that work-focused self-management interventions should be offered to individuals who report difficulty in work due to their fatigue.

The mean score for total and nocturnal pain indicated that participants in this study experienced mild-moderate pain. Initial analysis revealed that overall pain, and pain at night, was significantly associated with fatigue. This concurs with previous research which identified a relationship between pain and fatigue [12, 47]. In this study, those with high levels of fatigue had significantly higher total and nocturnal pain scores. This would indicate that effective fatigue management education should include pain management and joint protection strategies.

Interview participants reported using various strategies for managing their fatigue. These included pacing daily activities, modifying work routines, taking regular exercises, and taking a rest period during the day. Many of the strategies discussed were self-developed as participants in the qualitative part of this study reported a lack of formal education regarding fatigue in AS. Participants reported mixed reactions from healthcare professionals when they reported difficulty with fatigue which lead them to question health professionals' knowledge on this symptom. This has been identified in other studies of individuals with rheumatic diseases with fatigue management strategies mainly acquired through trial and error with little to no input from health professionals [15, 47, 48]. This indicates that further research is required in this area which could examine health professionals' knowledge and understanding of fatigue and strategies for managing fatigue. However, a participant in this study questioned whether the lack of formal education from health professionals on fatigue management was due to individuals not discussing fatigue with their health professionals. Further studies are needed to examine the extent of this finding with other individuals with AS. Qualitative studies are also needed to examine in more depth the educational needs of people with AS regarding their educational needs such as when is the optimal time for receiving this education and in what format.

How to measure fatigue in rheumatic diseases and the sensitivity of fatigue measures has received much attention in recent literature [26, 27]. In this current study, three different assessments were used to measure frequency and severity of fatigue: the FSS, BASDAI fatigue item (one-dimensional fatigue measures), and the MAF (a multidimensional assessment). The FSS indicated a high prevalence of fatigue with 64% of participants above the cut-off score. The BASDAI fatigue item had similar prevalence rates to the FSS, with 66% of participants scoring severe fatigue. In contrast to these two measures, the mean MAF-GFI score indicated a lower prevalence of fatigue with 38% of participants scoring above the cut-off score for high fatigue. Although quick to administer, single-dimensional assessments, such as the FSS, have been criticised for the limited information yielded thus leading them to inadequately measure this symptom [25]. Therefore, it is suggested that multidimensional fatigue assessments may provide a more accurate assessment of fatigue and provide information on how fatigue impacts on different types of activities which is important information for guiding suitable fatigue management interventions.

This study has provided information on the impact of AS-related fatigue on occupational participation. However, there are limitations to the study that could have impacted on the findings. The sample size was small and is representative only of the individuals who attend this particular AS clinic. The findings are also limited as data were only collected at one point in time whereas a longitudinal mixed method study would yield more comprehensive information on fluctuations in fatigue and in occupational participation over time. The qualitative aspect of this current study provided a general overview of impact of fatigue on occupational participation. However, the qualitative phase of a longitudinal mixed method study could use a phenomenological perspective to gain a more detailed and in-depth exploration of the experience of fatigue for individuals with AS.

## 6. Conclusion

This study explored the prevalence and impact of fatigue for individuals with ankylosing spondylitis. Three fatigue measures yielded different prevalence of rates in this sample thus indicating the need to ensure that the measure chosen to assess fatigue in individuals with AS provides the necessary information. For example, the FSS measure will provide information on severity of fatigue but is limited in measuring the impact of fatigue on daily activities.

There were significant differences between those with high and low levels of fatigue for disease activity, pain, frequency of occupational participation, and quality of life. Study participants identified fatigue as impacting specifically on work and social and leisure occupations. Fatigue management strategies were mainly self-taught indicating a need for formalised self-management support from relevant health professionals to reduce the impact of fatigue on productivity and leisure. Occupational therapists have specific skills and knowledge to provide these interventions.

## Figures and Tables

**Table 1:** Demographic profile.

Demographic variable	*n* (%) or mean (SD)
Total number of participants recruited	50
Age	46.5 years (SD ± 12.10)
Age range	
25-46 years	29 (58%)
47 and above	21 (42%)
Disease duration	14.50 years (SD ± 12.75)
Disease duration range	
1-14 years	28 (56%)
15 years and above	22 (44%)
Gender	
Men	36 (72%)
Women	14 (28%)
Marital status	
Single	15 (30%)
Married	32 (64%)
Separated/divorced/widowed	3 (6%)
Living situation	
Alone	6 (12%)
With someone	44 (88%)
Education levels	
Completed second level	21 (42%)
Completed third level courses	19 (38%)
Employment status	
Working	30 (60%)
Not working	20 (40%)

**Table 2:** Assessment mean values and standard deviations.

Assessment (score range)	Mean (SD) or *n* (%)
FSS (0-7)	4.2 (±1.6)
High fatigue scores (≥4)	64% (*n* = 32)
Low fatigue scores (≤3)	34% (*n* = 17)
BASDAI fatigue item (0-10)	5.6 (±2.4)
High fatigue scores (≥5)	33 (66%)
Low fatigue scores (≥4)	14 (28%)
MAF-GFI (0-50)	18.2 (±7.74)
High fatigue scores (≥21)	19 (38%)
Low fatigue scores (≤20)	31 (62%)
FAI total (0-45)	31.9 (±7.14)
Domestic (0-15)	11.5 (±3.45)
Leisure/work (0-15)	9.7 (±3.11)
Outdoors (0-15)	10.7 (±2.91)
BASDAI (0-10)	4.5 (±2.40)
PtGA	4.1 (±2.36)
BASFI (0-10)	4.1 (±2.52)
Pain (0-10)	
Total	4.0 (±2.37)
Nocturnal	3.4 (±2.64)
ASQoL (0-17)	7.0 (±4.92)
BAS-G (0-10)	4.6 (±2.52)

**Table 3:** Correlations between fatigue and study variables.

	FSS		MAF-GFI		BASDAI fatigue	
	Rho value	*p* value	Rho value	*p* value	Rho value	*p* value
FAI total	-0.16	0.27	-0.33	**0.018** ^∗^	-0.32	**0.031** ^∗^
BASDAI	0.32	**0.03** ^∗^	0.60	**0.001** ^∗∗^	0.72	**0.001** ^∗∗^
PtGA	0.21	0.18	0.55	**0.001** ^∗∗^	-0.33	**0.041** ^∗^
BASFI	0.34	**0.02** ^∗^	0.37	**0.012** ^∗^	0.45	**0.002** ^∗∗^
Pain						
Total	0.42	**0.004** ^∗∗^	0.54	**0.001** ^∗∗^	0.57	**0.001** ^∗∗^
Nocturnal	0.45	**0.002** ^∗∗^	0.56	**0.001** ^∗∗^	0.49	**0.001** ^∗∗^
ASQoL	0.56	**0.001** ^∗∗^	0.63	**0.001** ^∗∗^	0.36	**0.015** ^∗^
BAS-G	0.29	**0.050** ^∗^	0.64	**0.001** ^∗∗^	-0.72	**0.001** ^∗∗^

Significance level: ^∗^*p* < 0.05, ^∗∗^*p* < 0.001.

**Table 4:** Mann-Whitney *U* test: differences between high and low fatigue according to the MAF-GFI, in relation to activity levels, disease activity, functional ability, pain, and quality of life.

Questionnaire (score range)	MAF GFI (≥21) Median (range )	MAF GFI (≤20) Median (range)	*p* value
FAI (0-45)	30.0 (9-43)	35.0 (9-43)	**0.007** ^∗^
BASDAI (0-10)	6.5 (1-10)	3.1 (1-10)	**<0.001** ^∗∗^
PtGA (0-10)	6.0 (0-9)	3.0 (0-9)	**<0.001** ^∗∗^
BASFI (0-10)	6.00 (0-10)	3.0 (0-10)	**0.015** ^∗^
Pain (0-10)			
Total pain	6.0 (0-9)	3.0 (0-9)	**<0.001** ^∗∗^
Nocturnal pain	6.0 (0-9)	2.0 (0-9)	**<0.001** ^∗∗^
ASQoL (0-17)	11.0 (0-17)	4.0 (0-17)	**<0.001** ^∗∗^
BAS-G	7.5	3.0	**<0.001** ^∗∗^

Significance level: ^∗^*p* < 0.05, ^∗∗^*p* < 0.001.

**Table 5:** Demographics of interview participants.

	Age	Sex	Employment status	Marital status
Participant 11 (P11)	36	Male	Employed	Single
Participant 12 (P12)	48	Male	Employed	Married
Participant 15 (P15)	50	Male	Employed	Single
Participant 16 (P16)	48	Male	Employed	Married
Participant 18 (P18)	46	Male	Employed	Married
Participant 19 (P19)	48	Male	Employed	Married
Participant 02 (P2)	71	Male	Retired	Married
Participant 24 (P24)	48	Male	Employed	Married
Participant 25 (P25)	44	Male	Unemployed	Single
Participant 29 (P29)	65	Male	Retired	Single
Participant 34 (P34)	54	Male	Unemployed	Married
Participant 01 (P1)	41	Female	Unemployed	Married
Participant 13 (P13)	46	Female	Employed	Divorced
Participant 28 (P28)	36	Female	Employed	Single
Participant 03 (P3)	40	Female	Unemployed	Divorced
Participant 32 (P32)	49	Female	Unemployed	Married
Participant 35 (P35)	34	Female	Employed	Married
Participant 51 (P51)	38	Male	Employed	Married
Participant 52 (P52)	45	Male	Employed	Single

## Data Availability

The data used to support the findings of this study are available on request from the corresponding author.
